# Effects of Beta-Alanine Supplementation on Brain Homocarnosine/Carnosine Signal and Cognitive Function: An Exploratory Study

**DOI:** 10.1371/journal.pone.0123857

**Published:** 2015-04-14

**Authors:** Marina Yazigi Solis, Simon Cooper, Ruth M Hobson, Guilherme G. Artioli, Maria C. Otaduy, Hamilton Roschel, Jacques Robertson, Daniel Martin, Vitor S. Painelli, Roger C. Harris, Bruno Gualano, Craig Sale

**Affiliations:** 1 School of Physical Education and Sport, University of São Paulo, São Paulo, SP 05508–030, Brazil; 2 Biomedical, Life and Health Sciences Research Centre, Nottingham Trent University, Nottingham, NG11 8NS, UK; 3 LIM44, Faculty of Medicine, University of São Paulo, São Paulo, SP 05403–900, Brazil; 4 Junipa Ltd., Newmarket, Suffolk, UK; University of North Dakota School of Medicine and Health Sciences, UNITED STATES

## Abstract

**Objectives:**

Two independent studies were conducted to examine the effects of 28 d of beta-alanine supplementation at 6.4 g d^-1^ on brain homocarnosine/carnosine signal in omnivores and vegetarians (Study 1) and on cognitive function before and after exercise in trained cyclists (Study 2).

**Methods:**

In Study 1, seven healthy vegetarians (3 women and 4 men) and seven age- and sex-matched omnivores undertook a brain 1H-MRS exam at baseline and after beta-alanine supplementation. In study 2, nineteen trained male cyclists completed four 20-Km cycling time trials (two pre supplementation and two post supplementation), with a battery of cognitive function tests (Stroop test, Sternberg paradigm, Rapid Visual Information Processing task) being performed before and after exercise on each occasion.

**Results:**

In Study 1, there were no within-group effects of beta-alanine supplementation on brain homocarnosine/carnosine signal in either vegetarians (p = 0.99) or omnivores (p = 0.27); nor was there any effect when data from both groups were pooled (p = 0.19). Similarly, there was no group by time interaction for brain homocarnosine/carnosine signal (p = 0.27). In study 2, exercise improved cognitive function across all tests (P<0.05), although there was no effect (P>0.05) of beta-alanine supplementation on response times or accuracy for the Stroop test, Sternberg paradigm or RVIP task at rest or after exercise.

**Conclusion:**

28 d of beta-alanine supplementation at 6.4g d^-1^ appeared not to influence brain homocarnosine/carnosine signal in either omnivores or vegetarians; nor did it influence cognitive function before or after exercise in trained cyclists.

## Introduction

Carnosine is a dipeptide of the amino acids beta-alanine and L-histidine, which is synthesised endogenously. Synthesis appears limited by the availability of beta-alanine, which itself is synthesised within the body or obtained from the diet. The main dietary sources of beta-alanine are meat and fish containing carnosine and its methylated derivatives. Daily ingestion of carnosine and related peptides in current human diets ranges from <50 to >4000mg for those consuming vegetarian and very high meat-content diets [1]. It is known that most of the carnosine ingested in the diet is cleaved to its constituent amino acids in the enterocytes due to the presence of carnosinase in the jejunum [2]. In humans, any carnosine that makes it intact into the bloodstream is likely to be acted upon by carnosinase in plasma. This enzyme possesses a high activity and, as a consequence, circulating concentrations of carnosine in humans are remarkably low [3].

Carnosine is abundant in skeletal muscle [3] and, as a consequence of the pKa of the imidazole ring (value 6.83), participates in intracellular acid-base regulation during exercise [3]. Besides skeletal muscle, carnosine has been suggested to be metabolically relevant to others tissues, such as the brain and heart, where again it may act as a proton buffer. Other suggested roles of carnosine include acting as a membrane stabilizer, anti-oxidant, anti-glycating and anti-convulsant agent [4, 5].

The rate of carnosine synthesis in human skeletal muscle is limited by the availability of beta-alanine as a result of the low affinity of both the transporter and carnosine synthase for this, relative to the concentration of beta-alanine in plasma [3, 6]. It is likely that this holds true for other tissues where carnosine is synthesised *in situ*. Fasting plasma beta-alanine concentrations are very low; therefore the availability of beta-alanine is limiting to carnosine synthesis. However, beta-alanine ingested from food (usually less than 600mg per meal in the modern diet) results in a transient postprandial increase in the plasma beta-alanine concentration, which may exceed the Km of the transporter [3]. Accordingly, beta-alanine supplementation has been consistently shown to increase intracellular carnosine biosynthesis [3, 7]. However, these studies have focused on skeletal muscle synthesis and there is little information regarding the carnosine responses to beta-alanine supplementation in other tissues where it may be relevant, such as brain.

In muscle, carnosine synthesis is dependent upon the uptake of beta-alanine and L-histidine, as muscle cells cannot transport the intact dipeptide [8, 9]. In brain, carnosine transport into neuronal cells is possible via the specific transporter PepT2 [10, 11], although this is likely to be limited by the very low concentration of carnosine in blood. Likewise, beta-alanine transport into brain also appears to be possible via the beta-amino acid transporter [12], but again may be limited by the low concentrations of beta-alanine in the circulation [3]. In mammals, carnosine has been detected in different brain areas, where it may act as a neurotransmitter [13, 14]. In contrast, the only study assessing carnosine in human brain showed that little, if any, carnosine was present [15]. Despite this, human brain expresses an enzyme capable of synthesising both carnosine and homocarnosine [15] and is able to synthesise carnosine 3–5 times faster than homocarnosine [15]. Furthermore, the synthesis rate was dependent upon the availability of beta-alanine [15], suggesting that this might be the rate-limiting factor for carnosine synthesis even in the human brain. Given the very low circulating levels of beta-alanine, it is reasonable to assume that increased beta-alanine availability with supplementation could lead to increased levels of carnosine in the human brain. This is strengthened by experimental evidence demonstrating that beta-alanine supplementation significantly increases carnosine content in different brain areas in rats [14]. Similarly, it also seems reasonable to assume that diets low in beta-alanine, such as vegetarian diets, may lead to diminished brain carnosine.

There is increasing evidence to support potential therapeutic roles of carnosine [16]. It has been suggested that increased levels of carnosine or related dipeptides in brain may be of therapeutic relevance, particularly in conditions exacerbated by oxidative stress, including neurodegenerative diseases such as Alzheimer’s, Parkinson’s, epilepsy, and brain injury [17]. Recent studies have demonstrated that carnosine exerted a protective effect on the brain membrane in an experimental model of global ischemia [18] and ischemic brain injury [19]. Using a transgenic murine model, Herculano et al., [20] showed that oral carnosine supplementation was effective in preventing cognitive decline in Alzheimer disease, which could be attributed to the carnosine´s ability to inhibit beta-amyloid polymerisation and the citotoxic effects of beta-amyloid [21]. The antiglycating activity of carnosine may also be involved in the protection against Alzheimer disease [17]. In Parkinson patients, Boldyrev et al., [22] suggested that adjuvant treatment with dietary carnosine supplementation (1.5g·d^-1^) could ameliorate neurological symptoms (as assessed by the Unified Parkinson’s Disease Rating Scale), which was paralleled by a decrease in protein carbonyls in blood. Similarly, Fodovora et al., [23] showed that patients with chronic encephalopathy presented an improvement in cognitive aspects of information processing after 21 days of carnosine supplementation. In addition, it has been suggested that carnosine may ameliorate mental fatigue, memory, attention and motor speed in mentally stressful conditions [24, 25].

Moderate intensity exercise has been shown to have positive effects on cognitive function [26]. On the other hand, physically stressful conditions, such as fatiguing exercise, have been shown to have a detrimental effect on cognitive function [27, 28]. This has led to the speculation that increasing brain carnosine content could improve cognitive function, particularly in stressful conditions, such as following fatiguing exercise. Based on evidence supporting the role of beta-alanine supplementation in increasing brain carnosine in rodents, we also speculated that beta-alanine supplementation in humans could lead to increased brain carnosine, as inferred by the imidazole ring signal. In line with this, increased brain carnosine, achieved via beta-alanine supplementation, resulted in improved performance in behavioural tests in rats [14]. In humans, Gross et al., [29] reported that 5 weeks of beta-alanine supplementation at 3.2g·d^-1^ enhanced motivation and perceived state during high-intensity exercise. In contrast, a recent study did not show any positive effect of beta-alanine supplementation on a cognitive test in fatigued elite soldiers [28], although improved marksmanship following beta-alanine supplementation was shown. Notably, brain carnosine was not assessed in either of these human studies.

As beta-alanine can be rapidly transported into the brain [12] and accumulates in neuronal cells in a variety of mammals, including humans [30], and given that beta-alanine supplementation can increase carnosine in the cerebral cortex and hypothalamus in rats [14], we hypothesised that beta-alanine supplementation could increase brain carnosine, improving cognitive function in healthy humans following high-intensity exercise. To gather further knowledge on the role of dietary carnosine and beta-alanine intake on brain carnosine, including the responses to dietary supplementation, we also compared omnivores *vs*. vegetarians before and after beta-alanine supplementation. Carnosine may be measured in tissues by proton magnetic resonance spectroscopy (1H-MRS) along with free histidine and other compounds where histidine is in a nuclear magnetic resonance visible form. In muscle, signal output is assumed to be primarily due to carnosine. In brain, however, homocarnosine is thought to be the major contributor to the signal output, although, being a dipeptide of gamma-aminobutyric acid and histidine, homocarnosine is unlikely to change in response to beta-alanine supplementation. We proposed, therefore, to determine if the histidine imidazole ring detected by 1H-MRS (thereafter called homocarnosine/carnosine signal, given the theoretical contribution of both dipeptides to this spectrum) was changed when participants were supplemented with beta-alanine (Study 1, performed at the University of Sao Paulo, Brazil). In a separate study (Study 2, performed at Nottingham Trent University, UK), we investigated the effects of beta-alanine on cognitive function in trained cyclists before and after a 20km cycling time-trial.

## Methods

### Ethical approval

All experimental procedures described in both studies were approved by their respective local ethics committees (Ethics Committee from School of Medicine of University of Sao Paulo and Nottingham Trent University Ethical Advisory Committee), and were in accordance with the Helsinki Declaration of 1975, as revised in 1983.

### General Design and Supplementation Protocol

Prior to participation, all participants were fully informed of the risks and discomforts associated with the studies and all individuals provided written informed consent. The methods and results of each study are described separately to enhance clarity.

In both studies, the participants ingested two slow release tablets (CarnoSyn^SR^, Compound Solutions Inc., Vista, Calif., USA) each containing 800mg of beta-alanine (total dose per serving was 1.6g) four times per day, separated by 3–4 hour intervals, for a total daily dose of 6.4g. Beta-alanine tablets were tested by the manufacturer prior to release for the study and conformed to the label claim for beta-alanine content. All supplements were independently tested by HFL Sports Science (Fordham, Newmarket, UK) prior to use to ensure no contamination with steroids or stimulants according to ISO 17025 accredited tests.

### Study 1

#### Experimental Design and Participants

In an open label study, participants undertook brain 1H-MRS exams at baseline and after 4 weeks of beta-alanine supplementation. Seven healthy vegetarians (3 women and 4 men who had been on a vegetarian diet for at least 4 months) and 7 age- and sex-matched omnivores (3 women and 4 men) volunteered to participate. There were no significant differences in the demographic characteristics between vegetarians and omnivores (p > 0.05) (Table 1). The individuals who volunteered for participation were self-identified as lacto-ovo-vegetarians (n = 6), vegans (n = 1) or omnivores (n = 7), according to well-accepted criteria [31]. Afterwards, a systematic dietary intake analysis was performed by means of three 24-h food recalls undertaken on separate days (two weekdays and one weekend day) using a visual aid photo album of real foods, which ensured that the vegetarians´ diet was free of meat, including from fish. The participants verbally agreed to maintain similar dietary intake for the duration of the study. The compliance to beta-alanine supplementation was determined to be 100% according to the bottles that were returned to the research staff.

**Table 1:** Participants demographic characteristics and dietary beta-alanine intake—Study 1.

*Variable*	*Vegetarian (n = 7)*	*Omnivores (n = 7)*	*P*
***Age (y)***	27.33(4.18)	32.14(11.52)	0.333
***BMI (Kg/m*** ^***2***^ ***)***	24.74(2.52)	23.54(2.75)	0.426
***Schooling (y)***	16.33(2.42)	16.86(5.27)	0.819
***Dietary beta-alanine (mg/d)***	0	490.47(119.53)	0.001

Data are mean ± (1SD). No significant differences between vegetarians and omnivores were noted.

#### Magnetic Resonance Spectroscopy

**Fig 1:**
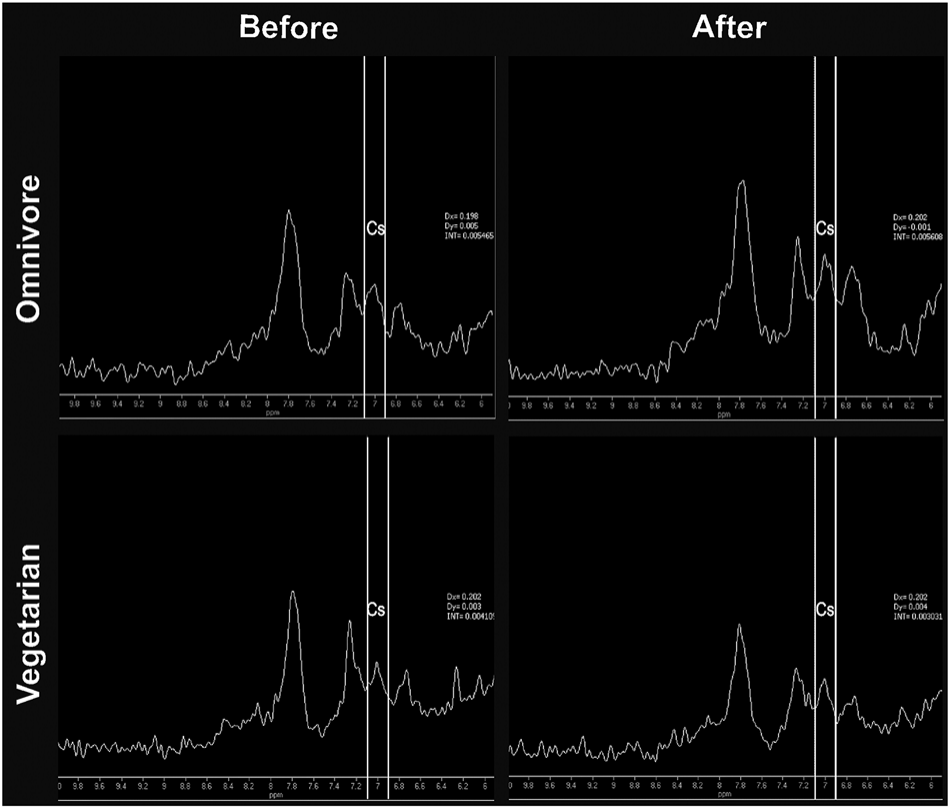
1H-MRS spectrum of an omnivore and a vegetarian subject before and after beta-alanine supplementation. Abbreviation: CS = homocarnosine/carnosine signal.

*In vivo* 1H-MRS of the posterior cingulate cortex (refer to ref. number [32] for details on voxel location) was acquired on a whole body 3.0T MRI scanner (Achieva Intera, Philips, Best, The Netherlands) using an eight-channel head coil. We chose to measure cingulate cortex due to its involvement in relevant cognitive function, such as processing, learning, and memory [33]. The spectroscopy sequence was a single voxel STEAM (voxel size 3x3x3cm^3^) with TE/TR = 10/1839 ms, spectral bandwidth of 2000Hz, 2048 sample points and 160 averages. The central frequency for acquisition was set to 8ppm. Metabolite quantification was performed on the Philips workstation using the Extended MR workspace interface. Before Fourier Transformation time domain signal was multiplied by a -1.5 Hz exponential function and followed by a 3Hz Gaussian filter. After residual water subtraction, an automatic zero and first order phasing procedure was applied. For quantification of the homocarnosine/carnosine signal (*i*.*e*., the signal corresponding to the histidine imidazole ring) we chose to quantify the peak at 7.05 ppm [34], since the other peak related to homocarnosine/carnosine at 8.02 ppm is very close to the much larger peak of the N-acetylaspartate amide group resonating at 7.9ppm. Metabolite concentrations were expressed relative to the creatine signal in the same spectrum without performing any correction for different relaxation properties of the metabolites. Water FWHM (frequency width at half maximum) values were 12±2 Hz on average (range from 10 to 18Hz). Quantification of homocarnosine/carnosine and creatine was obtained by numerical integration of the spectrum in the region of 6.9–7.1ppm and 2.8–3.1 ppm, respectively. The coefficient of variation of this measure was < 12% and the mean signal-to-noise ratio was 5.8. Fig 1 illustrates a representative 1H-MRS spectrum of an omnivore and a vegetarian subject before and after beta-alanine supplementation.

#### Statistical Methods

The effect of beta-alanine supplementation on the brain homocarnosine/carnosine signal signal in vegetarians and omnivores was assessed by a mixed model analysis (group x time interaction) using the SAS software (version 8.2; SAS Institute Inc., Cary, NC). The same statistical approach was used to assess any possible differences in the dietary intake of beta-alanine between vegetarians and omnivores across time. Data are reported as mean ± (1SD) and 95% interval confidence, unless otherwise stated. Statistical significance was accepted at p < 0.05.

### Study 2

#### Experimental Design and Participants

This was a randomised, double blind, placebo-controlled, parallel design experiment involving a familiarisation trial, two pre-supplementation trials and two post supplementation trials, all of which followed an identical protocol (S1 CONSORT Checklist).

**Fig 2:**
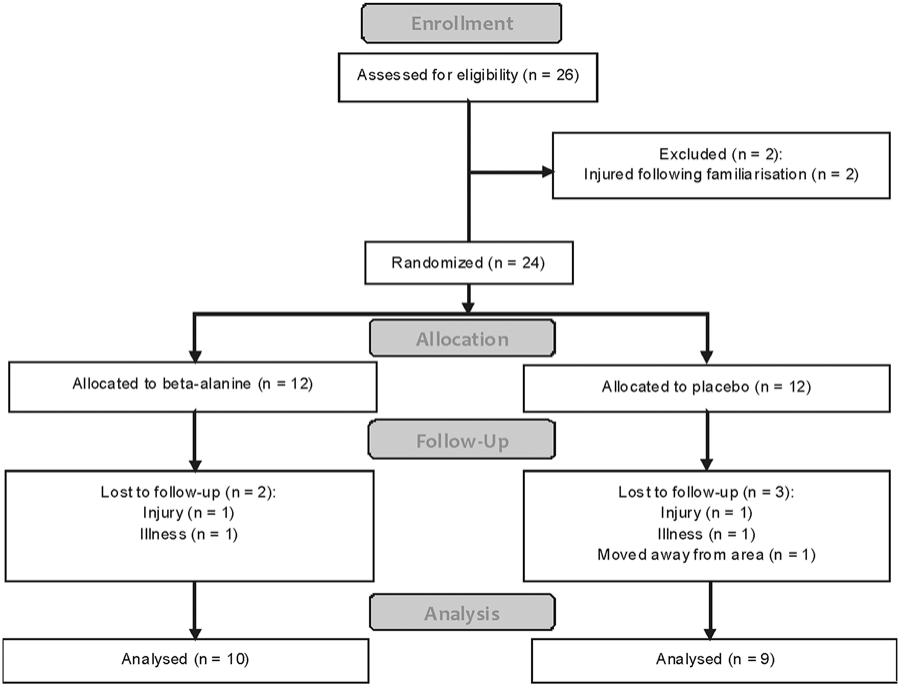
Flow diagram from study 2.

Twenty-six UK category 1 male cyclists volunteered to participate in the study and were randomly assigned to either a placebo (P; maltodextrin) or a beta-alanine (BA) supplementation group using the ABBA method described by Altman [35]. However, seven participants (4 from P and 3 from BA) withdrew from the study following completion of the baseline trials citing various reasons not associated with the study (Fig 2). As such, nineteen participants completed all trials; participant characteristics are presented in Table 2. Beta-alanine and placebo tablets were identical in appearance and were contained in identical white unlabelled pots. Identifying numbers were provided on each pot and an experimenter noted these before removing them from the pots and providing them to participants. The code was held by an experimenter not directly involved with data collection and this code was only broken after the completion of data collection. Supplementation logs were provided to each participant to ascertain compliance with the supplementation protocol. On average, compliance was 92% with beta-alanine and 89% with placebo.

**Table 2:** Participants demographic characteristics—Study 2.

*Variable*	*Beta-alanine supplemented*	*Placebo supplemented*	*P*
***Age (y)***	37(8)	32(6)	0.211
***Height (m)***	1.82(0.06)	1.81(0.04)	0.902
***Mass (kg)***	78.7(8.8)	80.4(8.3)	0.671

Data are mean ± (1SD). No significant differences between groups were noted.

Participants verbally confirmed prior to each laboratory visit that their health status had not changed and that they had not taken any supplement in the 3 months prior to the study and had not taken beta-alanine for at least 6 months prior to the study due to the long washout period for muscle carnosine. Participants verbally agreed to maintain similar levels of physical activity and dietary intake for the duration of the study during familiarisation and compliance with this request was verbally confirmed with participants prior to each testing session. Furthermore, dietary intake was assessed via a food record for 24 hours prior to the first experimental trial, which was then repeated prior to each trial. None of the cyclists were vegetarian and so would have ingested small amounts of dietary beta-alanine from the hydrolysis of carnosine and methyl derivatives of this in meat. This would typically be expected to vary daily between 50 to 500mg.

#### Testing Protocol

During preliminary testing, height (Seca, UK) and body mass (Seca, UK) were recorded before participants completed a full habituation test with the 20-Km cycling time-trial as described below. Each of the experimental trials were completed at least 4 h postprandial and participants had not completed vigorous exercise in the 24 h prior to each trial. Upon arrival to the laboratory, participants completed the cognitive function testing battery on a laptop in isolation in a private room while wearing noise cancelling ear defenders in order to reduce distraction.

After completing the pre-exercise cognitive function tests, the participants completed a self-paced 5-minute warm up followed by a 20-Km time trial; the methodological details of this aspect of the study (including test-retest reliability), and its performance data are presented elsewhere [36]. Upon completion of the 20-Km time trial, participants moved as quickly as possible back in to the isolation room to complete the battery of cognitive function tests for a second time. The average time taken from finishing the exercise test to starting the cognitive function tests was 151 ± 99 s.

#### Cognitive Function Tests

The battery of cognitive function tests was administered to participants via a laptop computer. The cognitive function tests consisted of the Stroop test, Sternberg paradigm and Rapid Visual Information Processing (RVIP) task. This testing battery has been successfully used in a study employing a similar population [27], with the tests demonstrating high test-retest reliability [37]. The tests were administered in the following order:

- Stroop Test: The Stroop test measures the sensitivity to interference and the ability to suppress an automated response [38] and is commonly used to assess selective attention. The Stroop test consists of two levels (baseline and complex) and is described in detail elsewhere [39]. In short, participants chose the correct response from a target and distractor presented on the screen, using the arrow keys. On the baseline level the target word matched the stimulus word on the centre of the screen, whereas on the complex level participants had to select the colour the stimulus word was written in, rather than the word itself. The variables of interest were the response times of the correct responses and the percentage of correct responses made.
- Sternberg Paradigm: The Sternberg Paradigm [40] is a test of working memory and has three levels. Each level used a different working memory load; one, three or five items. The full details of the Sternberg paradigm are provided elsewhere [39]. In short, on each level, participants had to select whether the stimulus on the screen matched one of the pre-determined target stimuli (a ‘3’ on the one item level and combinations of three or five letters on the three and five item levels respectively). The variables of interest were response times of the correct responses and the percentage of correct responses made.
- Rapid Visual Information Processing Task: The Rapid Visual Information Processing (RVIP) task is a continuous performance test lasting 5 min, requiring sustained attention and working memory. The RVIP task is described in detail elsewhere [27], but in short required participants to monitor a continuous stream of digits (using digits 2–9), presented at a rate of 100 digits/min (thus each digit was on the screen for 600 ms), to identify target sequences of 3 consecutive odd or even numbers (e.g. 3-9-5 or 2-6-4). The variables of interest were response times of correct responses and the percentage of correct responses made.

#### Statistical Methods

Data were analysed using SPSS (Version 18, SPSS Inc., Chicago, Il, USA). For both the pre- and post-supplementation testing points, data from the first of the two trials were used for familiarisation purposes with the second of the two trials being used for statistical analysis. To examine the effect of beta-alanine on the potential changes in cognitive function with exercise we conducted a three-way, supplement (beta-alanine or placebo) by time (pre and post supplementation) by exercise (pre and post exercise) analysis of variance (ANOVA), with repeated measures for time and exercise was conducted. To examine the effects of beta-alanine supplementation on cognitive function at rest, a two-way, supplement (beta-alanine or placebo) by time (pre and post supplementation) ANOVA, with repeated measures for time was conducted, using the pre-exercise data only. Finally, to check for differences between the groups at baseline, independent sample t-tests were conducted. All data are reported as mean±(1SD) unless otherwise stated and statistical significance was accepted at p < 0.05.

## Results

### Study 1

**Fig 3:**
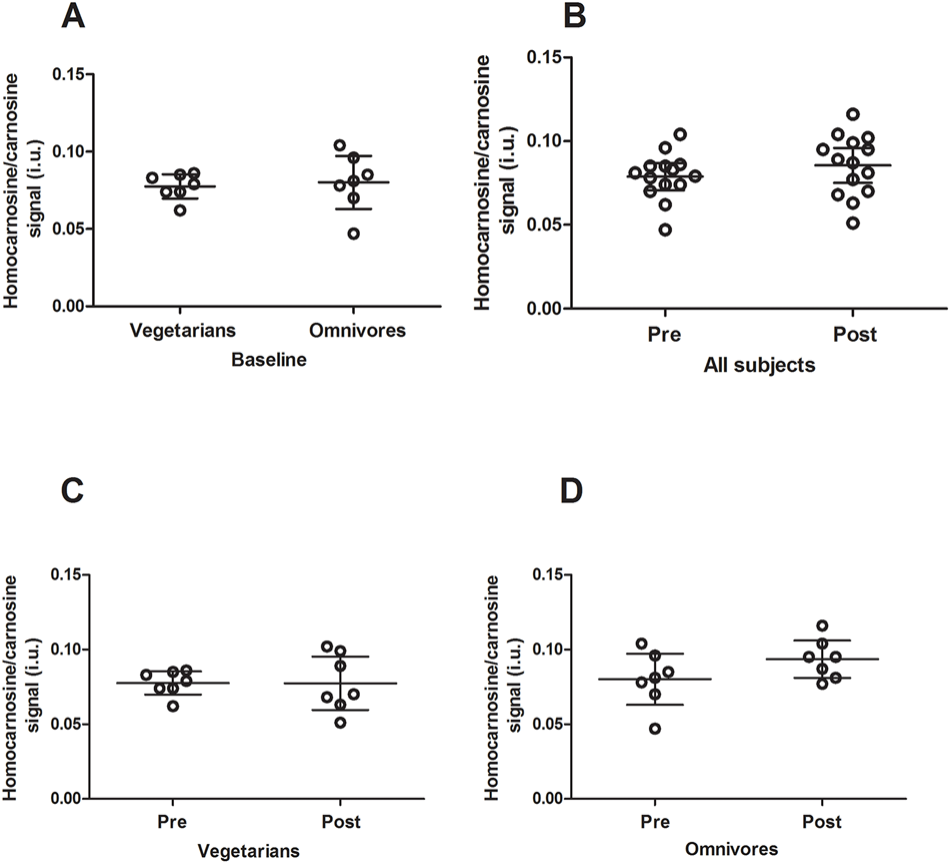
Homocarnosine/carnosine signal in vegetarians and omnivores before and after beta-alanine supplementation. Panel A: Homocarnosine/carnosine signal in vegetarians *versus* omnivores. Panel B: Effects of beta-alanine supplementation on brain homocarnosine/carnosine signal in all the subjects irrespective of their diet. Panel C: Effects of beta-alanine supplementation on homocarnosine/carnosine signal in vegetarians. Panel D: Effects of beta-alanine supplementation on homocarnosine/carnosine signal in omnivores. Data are expressed as individual data (circles), mean (central line) ± 95% confidence interval (lower and upper lines). Neither diet nor beta-supplementation significantly affected homocarnosine/carnosine signal.

As expected, beta-alanine intake in vegetarians was zero, whereas omnivores ingested 490.5 ± 119.5 mg/d. The homocarnosine/carnosine signal was comparable between vegetarians and omnivores (0.0996 ± 0.0134 *vs*. 0.1072 ± 0.0207 i.u.; p = 0.89) at baseline. In addition, no within-group effects of beta-alanine supplementation were observed upon the Cs/Cr ratio in vegetarians (p = 0.99), in omnivores (p = 0.27), or when data from both groups were pooled (p = 0.19) (Fig 3), suggesting no changes in brain homocarnosine/carnosine signal following beta-alanine supplementation. Similarly, no group by time interaction was detected (p = 0.27) (S1 Data).

### Study 2

For all cognitive tests the response times were first log transformed to normalise the distributions, which exhibited the right-hand skew typical of human response times (Table 3). According to task complexity, minimum and maximum response time cut-offs were set to exclude those responses that can be considered anticipations and delayed responses. As such, minimum response time cut-offs were set at 100 ms for the Stroop test and Sternberg paradigm and 250 ms for the RVIP task. Maximum response time cut-offs were set at 1300 ms (baseline level) and 2000 ms (complex level) for the Stroop test, 1200 ms (all levels) for the Sternberg paradigm and 1500 ms for the RVIP task. Only the response times of correct responses were used for response time analyses across all three cognitive tests.

**Table 3:** Response times and accuracy across the cognitive function tests.

			*Response Time (ms)*				*Accuracy (%)*			
*Test*	*Level*	*Supplement*	*Pre-Supplementation*		*Post-Supplementation*		*Pre-Supplementation*		*Post-Supplementation*	
			*Pre-exercise*	*Post-exercise*	*Pre-exercise*	*Post-exercise*	*Pre-exercise*	*Post-exercise*	*Pre-exercise*	*Post-exercise*
***Stroop***	*Baseline*	Beta-alanine	662 ± 192	637 ± 150	654 ± 157	578 ± 114	97.5 ± 3.5	98.5 ± 3.4	97.0 ± 5.4	97.5 ± 6.3
		Placebo	670 ± 142	630 ± 109	660 ± 129	608 ± 113	99.4 ± 1.7	98.3 ± 2.5	98.9 ± 3.3	99.4 ± 1.7
	*Complex*	Beta-alanine	922 ± 250	864 ± 205	867 ± 245	776 ± 166	95.8 ± 4.4	95.8 ± 4.4	96.0 ± 3.9	97.5 ± 4.2
		Placebo	946 ± 235	871 ± 222	853 ± 208	788 ± 187	98.1 ± 2.4	98.6 ± 1.8	97.2 ± 3.4	96.9 ± 3.9
***Sternberg***	*One letter*	Beta-alanine	462 ± 119	426 ± 124	421 ± 59	398 ± 56	97.9 ± 6.3	96.5 ± 6.3	97.2 ± 6.3	97.2 ± 3.3
		Placebo	444 ± 40	407 ± 43	448 ± 67	410 ± 55	99.3 ± 2.1	100.0 ± 0.0	98.6 ± 2.8	100.0 ± 0.0
	*Three letter*	Beta-alanine	539 ± 106	507 ± 102	520 ± 96	509 ± 86	97.9 ± 4.1	96.9 ± 4.7	96.2 ± 2.6	96.9 ± 2.7
		Placebo	538 ± 64	524 ± 68	527 ± 70	493 ± 65	98.3 ± 2.3	98.3 ± 2.3	98.3 ± 2.3	97.9 ± 5.2
	*Five letter*	Beta-alanine	637 ± 130	600 ± 118	614 ± 94	567 ± 92	96.5 ± 5.0	96.2 ± 2.6	96.2 ± 3.4	95.8 ± 3.8
		Placebo	631 ± 82	594 ± 102	648 ± 111	611 ± 109	97.9 ± 1.6	99.7 ± 1.0	98.6 ± 2.3	97.6 ± 2.1
***RVIP***	—	Beta-alanine	563 ± 160	502 ± 113	514 ± 125	500 ± 127	54.8 ± 24.6	59.7 ± 26.4	58.7 ± 26.1	64.5 ± 26.9
		Placebo	509 ± 83	472 ± 63	505 ± 72	466 ± 60	62.3 ± 21.7	69.0 ± 22.9	64.9 ± 24.1	73.1 ± 19.9

All data are mean ± standard deviation.

#### Stroop Test

Response Times: There was no difference in response times on either level of the Stroop test between the groups prior to supplementation (baseline level, p = 0.916; complex level: p = 0.829). On both levels of the Stroop test, participants responded quicker post-exercise when compared to pre-exercise (main effect of exercise: baseline level, 613 ± 28 ms vs. 661 ± 36 ms, p = 0.006; complex level, 825 ± 43 ms vs. 897 ± 53 ms, p < 0.0005). However, supplementation with beta-alanine did not influence the effects of exercise on response times on either level of the Stroop test (supplement by time by exercise interaction: baseline level, p = 0.228; complex level, p = 0.451). Furthermore, there was no effect of beta-alanine supplementation on response times on either level of the Stroop test at rest (supplement by time interaction: baseline level, p = 0.929; complex level, p = 0.314).

Accuracy: There was no difference in accuracy on either level of the Stroop test between the groups prior to supplementation (baseline level, p = 0.151; complex level: p = 0.216). There was also no change in accuracy following exercise on either level of the Stroop test (main effect of exercise: baseline level, p = 0.644; complex level, p = 0.329). Supplementation with beta-alanine did not influence the effects of exercise on accuracy on either level of the Stroop test (supplement by time by exercise interaction: baseline level, p = 0.407; complex level, p = 0.303). There was also no effect of beta-alanine supplementation on accuracy on either level of the Stroop test at rest (supplement by time interaction: baseline level, p = 0.972; complex level, p = 0.539).

#### Sternberg Paradigm

Response Times: There was no difference in response times on any level of the Sternberg paradigm between the groups prior to supplementation (one letter level, p = 0.684; three letter level, p = 0.981; five letter level, p = 0.910). On all levels of the Sternberg paradigm, participants responded quicker post-exercise when compared to pre-exercise (main effect of exercise: one letter level, 410 ± 16 ms vs. 444 ± 17 ms, p < 0.0005; three letter level, 508 ± 19 ms vs. 531 ± 19 ms, p = 0.008; five letter level, 593 ± 23 ms vs. 633 ± 23 ms, p < 0.0005). However, supplementation with beta-alanine did not mediate the effects of exercise on response times on any level of the Sternberg paradigm (supplement by time by exercise interaction: one letter level, p = 0.642; three letter level, p = 0.177; five letter level, p = 0.805). Furthermore, there was no effect of beta-alanine supplementation on response times on any level of the Sternberg paradigm at rest (supplement by time interaction: one letter level, p = 0.156; three letter level, p = 0.767; five letter level, p = 0.283).

Accuracy: There was no difference in accuracy on any level of the Sternberg paradigm between the groups prior to supplementation (one letter level, p = 0.523; three letter level, p = 0.828; five letter level, p = 0.431). There was also no change in accuracy following exercise on any level of the Sternberg paradigm (main effect of exercise: one letter level, p = 0.758; three letter level, p = 0.765; five letter level, p = 0.958). Supplementation with beta-alanine did not influence the effects of exercise on accuracy on any level of the Sternberg paradigm (supplement by time by exercise interaction: one letter level, p = 0.720; three letter level, p = 0.463; five letter level, p = 0.439). There was also no effect of beta-alanine supplementation on response times on any level of the Sternberg paradigm at rest (supplement by time interaction: one letter level, p = 0.951; three letter level, p = 0.404; five letter level, p = 0.645).

#### RVIP Task

Response Times: There was no difference in response times on the RVIP task between the groups prior to supplementation (p = 0.382). Participants responded quicker post-exercise when compared to pre-exercise (485 ± 22 ms vs. 523 ± 26 ms respectively, main effect of exercise: p < 0.0005), but beta-alanine supplementation did not influence the effects of exercise on response times to the RVIP task (supplement by time by exercise interaction, p = 0.126). There an effect of beta-alanine supplementation on response times to the RVIP task at rest (supplement by time interaction: p = 0.173).

Accuracy: There was no difference in accuracy on the RVIP task between the groups prior to supplementation (p = 0.489). Participants achieved a greater percentage of correct responses post-exercise when compared to pre-exercise (66.7 ± 5.5% vs. 60.3 ± 5.4%, main effect of exercise: p = 0.009). However, supplementation with beta-alanine did not mediate the effects of exercise on accuracy of the RVIP task (supplement by time by exercise interaction, p = 0.906), nor was there an effect of beta-alanine supplementation on accuracy on the RVIP task at rest (supplement by time interaction: p = 0.803).

## Discussion

To our knowledge, this is the first study to assess the effect of beta-alanine supplementation on brain homocarnosine/carnosine signal in humans and the mediating effects of beta-alanine supplementation on the acute effects of exercise on cognitive function. Our main findings were that 4 weeks of beta-alanine supplementation did not change the brain homocarnosine/carnosine signal in either vegetarian or omnivorous healthy individuals, nor was there any effect of beta-alanine supplementation on cognitive function in athletes at rest or following exercise.

Carnosine is metabolically relevant to brain cells, since it acts as a neurotransmitter and endogenous neuroprotective agent [41]. Studies have suggested that an elevation of carnosine levels (or related dipeptides) in brain may improve cognitive function [23], mental fatigue [24] and memory [25], as well as ameliorate symptoms related to neurodegenerative diseases (*e*.*g*., Alzheimer’s and Parkinson’s diseases), epilepsy, and brain injury [42].

Beta-alanine supplementation is capable of increasing the carnosine content of skeletal muscle [3] and beta-alanine appears to be rapidly transported in to the brain via the beta-amino acid transporter [12]. Moreover, beta-alanine supplementation has been shown to significantly increase carnosine content in different brain areas in rats [14]. Taken together, these findings led to the hypothesis that beta-alanine supplementation might be capable of increasing brain carnosine concentrations in humans. It should be noted that there is scant evidence supporting the presence of carnosine in the human brain. The only study available, which assessed carnosine in the brain of human cadavers, revealed little, if any, carnosine in brain regions other than olfactory bulb [15]. However, the same study showed that *in vitro* carnosine synthesis in samples of the human temporal cortex was dependent upon the availability of beta-alanine, and the enzyme that synthesises both homocarnosine and carnosine in human brain supernatants forms carnosine 3–5 times as rapidly as it forms homocarnosine [15]. Collectively, these data allowed us to speculate that orally ingested beta-alanine availability could be the limiting factor for carnosine synthesis in brain. In line with this, we also hypothesised that diet could influence homocarnosine/carnosine signal given that vegetarians (who do not consume beta-alanine in their diet) have lower skeletal muscle carnosine content when compared to omnivores [43].

The results of the present study (Study 1), however, showed that beta-alanine supplementation did not alter the homocarnosine/carnosine signal in the brain spectrum, suggesting that this hypothesis does not hold true. Thus, these data suggest that brain carnosine synthesis does not rely upon beta-alanine uptake from the bloodstream in humans, contrasting previous *in vitro* [44] and animal studies [14].

Despite the growing number of studies suggesting that carnosine and/or beta-alanine supplementation may improve cognitive aspects in a variety of populations [25, 45], we did not observe any positive effect of beta-alanine supplementation upon cognitive measurements either at rest or following exercise. In agreement with our results, Hoffman et al. [28] did not observe any improvement in cognitive function in fatigued soldiers. The possible elevation in brain carnosine content via supplementation has been considered the most plausible mechanism to explain improvements in cognitive function observed in these studies. Our data do not support this hypothesis, given that the data from Study 2 are in line with the lack of changes in the homocarnosine/carnosine signal shown in Study 1. It should be noted, that other factors, such as participant characteristics (*e*.*g*., healthy, diseased, athletes, younger, older individuals), the cognitive tests employed (memory, attention, time-to-reaction, etc), the combination with other conditions that may affect mental performance (*e*.*g*., exercise, neurodegenerative disease, psychiatric disorders), the type of supplement administered and its different protocols (*e*.*g*., beta-alanine, carnosine; short- and long-term protocols), could also partially explain the differences in the outcomes between these investigations.

In order to assess brain carnosine, we used 1H-MRS, a method that has been used to determine muscle carnosine concentrations by allowing the detection of the signal resulting from the protons in the histidine imidazole ring [46, 47]. In brain, the same 1H-MRS signal, usually referred as Cs, is often attributed to homocarnosine (gamma-aminobutyryl-L-histidine) [48], a dipeptide analogue to carnosine. This comprises most of the Cs signal [25]. Although there is experimental data confirming the presence of carnosine in the brain of mammals [49], where it is thought to play a role in neurotransmission and neuroprotection [18], the signal obtained by the 1H-MRS technique is the undistinguishable result of both carnosine and homocarnosine. Therefore, an important limitation of this study is that, due to the influence of homocarnosine and other possible macromolecules containing histidine upon the signal at 7.05ppm, one might argue that the brain carnosine must be substantial in order to be detectable. To date, however, it is unknown whether diet or beta-alanine supplementation can influence brain carnosine concentrations in humans. Nonetheless, considering that a ~400 uM increase in brain histidine was shown to be detectable [50], it is plausible to assume that increases in carnosine concentration within the micromolar range would be detected by the 1H-MRS method. In order to further explore these questions, future studies should search for methodological refinements to improve both sensitivity and specificity of brain carnosine detection via 1H-MRS, perhaps by using larger voxel sizes, a higher number of averages (resulting in longer acquisition times) [50], and special preparation pulses to minimize the contribution of macromolecules to the homocarnosine/carnosine signal [5].

It is important to also emphasise that our 1H-MRS results are limited to the brain region assessed in this study (*i*.*e*., posterior cingulate cortex), which is part of the limbic system and has been related to emotions as well as cognitive function (*e*.*g*., processing, learning, and memory) [33]. The fact that the posterior cingulate cortex is implicated in cognitive function was the reason for choosing this region to measure in our study. Although cerebral cortex and other brain areas (*e*.*g*., hypothalamus and olfactory bulb) are susceptible to elevations in carnosine following beta-alanine supplementation, at least in rodents, we cannot rule out an effect of beta-alanine supplementation on carnosine in other brain areas that were not assessed in our study. It seems unlikely, however, that any putative change in carnosine in other areas will result in beneficial effects on cognition. It is also possible that beta-alanine supplementation can somehow affect brain metabolism/function by increasing brain beta-alanine content in its free form rather than as a dipeptide (*i*.*e*., carnosine). There is some speculation that beta-alanine may act as a neurotransmitter in its own right, based on data indicating that this occurs naturally in the brain, is released by electrical stimulation through a Ca^2+^ dependent processes, has binding sites, and inhibits neuronal excitability. Unfortunately, to date, there is no empirical evidence from humans to prove this assumption.

In conclusion, this study showed no changes on posterior cingulate cortex homocarnosine/carnosine signal after beta-alanine supplementation in healthy participants, regardless of their diets. This supports the lack of an effect of beta-alanine supplementation on cognitive function in trained cyclists before or after exercise. Taken together, these findings do not support the hypothesis that beta-alanine supplementation can promote beneficial effects on cognitive performance measured in association with exercise.

## Supporting Information

S1 CONSORT Checklist CONSORT Checklist for study 2. (DOCX)

S1 Data Dataset from study 1. (XLSX)

S2 Data Dataset from study 2. (XLSX)
