# Supplementary material for: Effects of Beta-Alanine Supplementation on Brain Homocarnosine/Carnosine Signal and Cognitive Function: An Exploratory Study
Source: PLoS One. 2015 Apr 14;10(4):e0123857. doi: 10.1371/journal.pone.0123857 (PMC4397072; doi:10.1371/journal.pone.0123857)
Supplement: S1 CONSORT Checklist — (DOCX) [file pone.0123857.s001.docx]

US 5,965,596: Methods and Compositions for Increasing the Anaerobic Working Capacity in Tissues; US 6,172,098: Methods and Compositions for Increasing the Anaerobic Working Capacity in Tissues; US 6,426,361: Methods and Compositions for Increasing the Anaerobic Working Capacity in Tissues; US 6,680,294: Methods and Compositions for Increasing the Anaerobic Working Capacity in Tissues; US 7,504,376: Methods and Compositions for Increasing the Anaerobic Working Capacity in Tissues; US 7,825,084: Methods and Compositions for Increasing the Anaerobic Working Capacity in Tissues; US 8,067,381: Methods and Compositions for Increasing the Anaerobic Working Capacity in Tissues; US 8,129,422: Methods and Compositions for Increasing the Anaerobic Working Capacity in Tissues; US 8,470,865: Methods and Compositions for Increasing the Anaerobic Working Capacity in Tissues; Canada 2,263,184: Methods and Compositions for Increasing the Anaerobic Working Capacity in Tissues; Canada 2,521,987: Methods and Compositions for Increasing the Anaerobic Working Capacity in Tissue; China ZL97198602.9: Methods and Compositions for Increasing the Anaerobic Working Capacity in Tissue; Europe EPO918469: Methods and Compositions for Increasing the Anaerobic Working Capacity in Tissue; Japan 4439019: Methods and Compositions for Increasing the Anaerobic Working Capacity in Tissue; Japan 5475396: Methods and Compositions for Increasing the Anaerobic Working Capacity in Tissue; US 8,329,207: Compositions and Methods for the Sustained Release of Beta-Alanine; US 8,394,402: Compositions and Methods for the Sustained Release of Beta-Alanine; US 8,496,958: Compositions and Methods for the Sustained Release of Beta-Alanine; Canada 2,621,365: Compositions and Methods for the Sustained Release of Beta-Alanine; Korea 10-1190791: Compositions and Methods for the Sustained Release of Beta-Alanine; Korea 10-1275660: Compositions and Methods for the Sustained Release of Beta-Alanine; Japan 5336180: Compositions and Methods for the Sustained Release of Beta-Alanine
